# Transitioning Adolescents and Young Adults with Type 1 Diabetes Mellitus in Italy: A Scoping Review

**DOI:** 10.3390/children13020248

**Published:** 2026-02-10

**Authors:** Valentina Vanzi, Ilaria Campagna, Fabiola Spina, Adele Passaro, Federica Cancani, Annalisa Deodati, Orsola Gawronski, Emanuela Tiozzo, Immacolata Dall’Oglio

**Affiliations:** 1 Department of Biomedicine and Prevention, University of Rome Tor Vergata, 00133 Rome, Italy; 2 Clinical Area of University Hospital Pediatrics, Bambino Gesù Children’s Hospital, IRCCS, 00165 Rome, Italy; ilaria.campagna@opbg.net (I.C.); fabiola.spina@opbg.net (F.S.); adele.passaro@opbg.net (A.P.); federica.cancani@opbg.net (F.C.); 3 JBI Italy Evidence—Based Practice and Health Research Centre, 00136 Rome, Italy; 4 Endocrinology & Diabetes Unit, Bambino Gesù Children’s Hospital, IRCSS, 00165 Rome, Italy; annalisa.deodati@opbg.net; 5 Department of Systems Medicine, University of Rome Tor Vergata, 00133 Rome, Italy; 6 Professional Development, Continuing Education and Research Service, Bambino Gesù Children’s Hospital, IRCCS, 00165 Rome, Italy; orsola.gawronski@opbg.net; 7 Research Unit Science of Healthcare Professions, Bambino Gesù Children’s Hospital, IRCCS, 00165 Rome, Italy; emanuela.tiozzo@opbg.net

**Keywords:** transition of care, adolescent, young adults, type 1 diabetes mellitus, Italy, scoping review

## Abstract

**Background/Objectives:** Worldwide, Type 1 diabetes mellitus (T1DM) in youth represents a growing public health concern, and Italy is among the countries with the highest incidence in the pediatric population. The transition from pediatric to adult care is a vulnerable period associated with increased risks of acute complications and long-term morbidity. This scoping review aimed to map the available Italian evidence on healthcare transition in adolescents and young adults (AYAs) with T1DM, addressing five key areas: characteristics of the transition process and involved populations, emotional and psychological experiences, the role of technology, existing transitional care models and related outcomes, and assessment criteria and tools for transition readiness. **Methods:** This review followed the JBI methodology and included studies focused on Italian AYAs (aged 10–24 years) with T1DM. Study selection was documented using the PRISMA flow chart. **Results:** Twenty studies were included. The evidence revealed a heterogeneous and inconsistently implemented transition landscape. Several structured transition projects were identified, differing in multidisciplinary team composition, organization, and outcome evaluation. Emotional distress, fear of separation from pediatric providers, and variable satisfaction with transition experiences were commonly reported. Adoption of technologies increased over time and was associated with improved clinical outcomes, although overall uptake remained suboptimal. Importantly, no Italian-validated tools for assessing transition readiness were identified. **Conclusions:** Transitional care for Italian AYAs with T1DM is increasingly recognized but remains insufficiently standardized and evaluated. Future research should prioritize multicenter studies, stratified analyses, and the development of culturally validated readiness assessment tools to support effective and individualized transitions.

## 1. Introduction

The prevalence of chronic conditions in pediatric and adolescent populations is increasing worldwide, driven by both rising incidence linked to lifestyle and environmental factors and improved survival due to advances in medical, surgical, and rehabilitative care. Consequently, the transition from pediatric to adult healthcare services has become a global priority. Diabetes is a major global public health concern, with early-onset forms of both type 1 and type 2 diabetes emerging as significant chronic conditions in the pediatric population [1,2]. Children and adolescents with type 1 diabetes mellitus (T1DM), the predominant form of diabetes diagnosed in childhood, face a heightened risk of early-onset complications, comorbid conditions, and increased mortality [3]. The overall burden of diabetes is further intensified by its steadily increasing prevalence worldwide, posing substantial challenges to individuals, families, and healthcare systems [2]. Globally, limited research is available on the epidemiology of T1DM, and its exact prevalence remains uncertain. Many countries lack national registries, and in those where registries do exist, data completeness is often questionable due to underreporting and underestimated case identification [1]. It is estimated that worldwide, approximately 9 million people are affected by T1DM, of whom 6% are in the 0–14 age group, 35% are between 15 and 39 years old, and the remaining 59% are aged 40 years or older [4]. Over a quarter (27%) of the worldwide cases of T1DM are registered in Europe [5]. Western Europe constitutes a particularly relevant region for assessing the burden of these diseases in youth due to the relative epidemiological homogeneity across countries. However, despite this comparability, major challenges persist in accurately evaluating the impact of diabetes in this age group, primarily due to fragmented data collection systems and a lack of standardized adolescent-specific indicators [6]. The Global Burden of Diseases 2019 (GBD 2019) provides a standardized framework to assess incidence, prevalence, and disability burden of T1DM and T2DM among individuals aged between 10 and 24 years across countries and over time. Its findings show a clear and consistent increase in all epidemiological indicators for both conditions in Western Europe [6]. Consistent with international evidence [7,8,9], epidemiological data from Italy also indicate a rising prevalence of diabetes in the pediatric population [10,11]. The epidemiological trend of pediatric diabetes in Italy has been substantially supported by the implementation of international initiatives such as DIAMOND and EURODIAB [8], which promoted the establishment of multiple national registries. These registries have played a pivotal role in enhancing knowledge on the incidence and distribution of T1DM in the Italian pediatric population, leading to the publication of high-impact international studies. Most of these registries have focused on newly diagnosed cases in the 0–14-year age group [12]. In response to the growing interest among Italian research groups in T1DM epidemiology, the Italian Registry for Type 1 Diabetes (Registro Italiano per il Diabete di tipo 1, RIDI) was established in 1997 as a collaborative initiative between the Italian Society of Diabetology (SID) and the Italian Society for Pediatric Endocrinology and Diabetology (SIEDP) [12,13]. The main objectives of RIDI were to coordinate existing incidence registries nationwide for the pediatric age group and to promote the activation of registries in regions that were not yet under epidemiological surveillance [12].

In Italy, the incidence and prevalence of T1DM show considerable regional variability, with higher rates typically observed in the northern regions compared to the south. An exception is Sardinia [14,15], where the incidence of T1DM has nearly doubled over the past 20 years and is currently among the highest reported worldwide: between 2019 and 2022, 512 patients aged 0–14 years were diagnosed with T1DM in Sardinia. The overall incidence rate was 73.9 per 100,000 person-years [16]. In the epidemiological study conducted by Bruno and colleagues (2010) the incidence of T1DM in Italy was 12.26 per 100,000 person-years, higher in boys than in girls [15]. A significant annual increase of 2.94% was also reported. Maffeis and colleagues (2021) analyzed T1DM prevalence and incidence rates in the pediatric population of the Veneto Region between 2015 and 2020, and they calculated that the overall prevalence of diagnosed diabetes in the age group of 0–18 increased over time from 149.4/100,000 in 2015 to 158.9/100,000 in 2020, with similar trends in males and females [17]. Accordingly, these authors stated that Veneto Region can be defined as a high-risk area for pediatric T1DM [17].

Monitoring trends in T1DM incidence and prevalence is essential to inform targeted strategies for early diagnosis, particularly in high-risk populations. Overall, these findings underscore a growing health challenge among young populations across the region, with implications for healthcare systems, prevention strategies, and long-term disease management [6,9]. As the number of children and adolescents diagnosed with T1DM continues to rise, the population of young people requiring transition from pediatric to adult healthcare services is also expanding significantly. This transition represents a critical phase in the continuum of care for youth with diabetes, as it involves not only a change in the healthcare setting but also a shift in responsibility for managing a complex and demanding treatment regimen [18,19,20]. Navigating this period can be particularly challenging, as young individuals must assume responsibility for daily diabetes self-management tasks, such as monitoring blood glucose, adjusting insulin dosages, and making informed decisions regarding nutrition, physical activity, and lifestyle. In addition, they must develop the skills necessary to engage with the adult healthcare system, including scheduling appointments, communicating with healthcare professionals, and dealing with insurance providers, tasks that were previously managed by their caregivers, especially during the initial stages of diagnosis when education is often directed primarily at parents [21]. This transitional phase is further complicated by the broader developmental changes that adolescents typically undergo, including shifts in education, employment, living arrangements, and personal relationships. These simultaneous transitions can contribute to instability and increased vulnerability [22]. As adolescents grow, both pediatricians and parents may be perceived as overprotective [23,24]. While the transition from a family-centered pediatric clinic to an adult care setting can foster autonomy, it may also generate anxiety and reduce adherence to follow-up.

Adult care units tend to focus on acute medical issues and may be less equipped to address the broader psychosocial needs of young patients. Additionally, families often develop a strong sense of security within the long-standing relationship with the pediatric team, which can contribute to resistance to the transition process [23,25]. Evidence shows that this period is associated with increased health risks. Many adolescents disengage from diabetes care during transition, with approximately one in three discontinuing regular follow-up or support services [26]. Poorly managed transitions are linked to adverse outcomes such as worsening glycemic control, increased hospitalizations, greater use of emergency services, and heightened emotional distress for both patients and families [22,27,28,29,30]. The American Diabetes Association (ADA) has emphasized that pediatric diabetes care cannot simply be extrapolated from adult models [31].

In this context, “transitional care” refers to structured interventions designed to support young people as they move from child-centered to adult-oriented healthcare systems [32]. This approach, originally defined by Blum et al. (1993) as “the purposeful, planned movement of adolescents and young adults with chronic physical and medical conditions from child-centered to adult-oriented health care systems” highlights the importance of a deliberate and coordinated process to ensure continuity of care and minimize risks [33]. Without effective transitional support, young adults with diabetes face a significantly increased likelihood of long-term complications and poor health outcomes.

The Italian healthcare system is founded on a universal, publicly funded National Health Service (Servizio Sanitario Nazionale, SSN), which ensures free and equitable access to healthcare for all residents. Diabetes care is delivered through specialized pediatric and adult diabetes centers, primarily within hospital-based outpatient services. Pediatric diabetes care is generally provided by multidisciplinary teams within pediatric centers. The transition from pediatric to adult care in Italy is commonly associated with reaching the age of 18 years, which represents the legal threshold between pediatric and adult healthcare services. However, there is no national legislation or standardized policy mandating a fixed age or procedure for transition. As a result, the timing and organization of transition remain highly variable and are largely determined by local practices, regional healthcare organization, and individual center policies. In some contexts, transition may occur gradually over a broader age range, while in others, it may take place abruptly at legal adulthood.

Grounded in current epidemiological insights and evidence-based considerations, and acknowledging the variability of regional healthcare organization and sociocultural settings, this scoping review was designed to map the body of evidence concerning the transition of youth with T1DM within the Italian context. Specifically, the sub-questions of this scoping review were: (1) to outline the healthcare transition process in Italy and the profiles of AYAs involved; (2) describe Italian AYAs’ experiences, emotional challenges and psychological disorders related to the transition process; (3) assess how technology and medical devices influence the transition process; (4) identify strategies, models or interventions for transitional care and related outcomes of transition process in Italy, and (5) review the assessment criteria and availability of Italian tools for evaluation of transition readiness.

## 2. Materials and Methods

A scoping review was conducted in accordance with the JBI methodology for scoping reviews and reported in adherence to the PRISMA-ScR guidelines [34,35]. The search strategy aimed to identify both published and unpublished studies. A three-step search strategy was used in this review, based on the Population–Concept–Context (PCC) framework: population (AYAs with T1DM), concept (transition from pediatric to adult care), and context (the Italian healthcare system). Initially, a broad search was conducted in both CINAHL and MEDLINE databases to analyze the text words contained in the title and abstract and the index terms used to describe the papers. A second search based on all the identified keywords and index terms was undertaken across all of the selected databases. Thirdly, the reference lists of all the included papers were scanned to look for publications that had not been identified previously. The search had no restrictions on date of publication, while the language criteria limited the selection to studies written in English and Italian to ensure their relevance to the Italian healthcare context. We searched MEDLINE (Pubmed), CINAHL (EBSCOhost), SCOPUS (Elsevier), Web of Science (Clarivate), Embase (Elsevier), and the Cochrane library and Google Scholar. The search for unpublished studies included OpenGrey. The main keywords were “transition”, “transition to adult care”, “transitional care”, “adolescent”, “young adult”, “youth”, “diabetes mellitus” and “insulin-dependent diabetes”. Boolean operators, NOT, AND, OR, were also used to narrow or widen the search. The study selection was conducted in May 2025. An example of the search strategy for MEDLINE (Pubmed) can be found in the Supplementary Materials.

**Figure 1:**
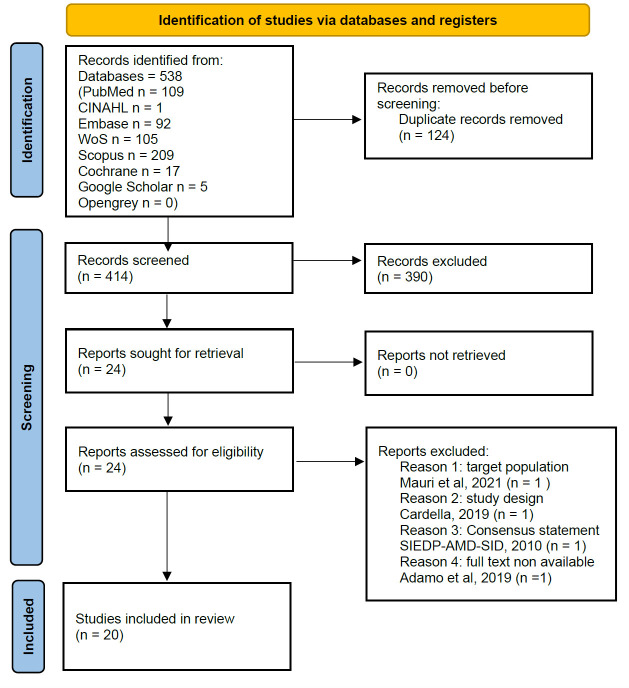
PRISMA flow chart of included studies [38,39,40,41].

A study was eligible for review if it reported data on AYAs, typically aged 10–24, according to the WHO definition [36] with T1DM undergoing healthcare transition in the Italian context. Studies reporting outcomes at older ages were eligible provided that the transition process occurred within the defined AYA age range. Eligible studies included quantitative, qualitative, and mixed-methods designs. Grey literature, conference abstracts, and policy documents were also considered when relevant information was provided. Studies were deemed non-eligible if they involved populations not meeting the AYA definition (10–24 years), focused exclusively on non-T1DM chronic conditions or failed to disaggregate data for T1DM, were conducted outside the Italian healthcare context, and did not address the transition from pediatric to adult healthcare services. Commentaries, editorials, letters to the editor, and opinion pieces without empirical data were also excluded. The Preferred Reporting Items for Systematic reviews and Meta-Analyses (PRISMA) flow chart, updated in 2020, was used to describe the study selection process: firstly, duplicate records were identified and removed [37]. Secondly, titles and abstracts were screened independently by two authors. Then, full texts of potentially eligible studies were read to determine if the studies were eligible. In case of discrepancies between the two primary reviewers, disagreements were first discussed in a consensus meeting using predefined decision rules (population, concept, context alignment and minimum data sufficiency). If consensus could not be reached, a third senior reviewer independently assessed the record and adjudicated the last decision. Finally, through the data extraction synthesis table, the following items were reported: authorship and year of publication, Italian geographical area where the study was conducted, type of article, study design, aim of the study, sample size and characteristics of the population, main findings or results. Each study was then mapped back to the relevant scoping review sub-question framework to extract data addressing the specific research objective. The PRISMA flow chart of this scoping review is shown in Figure 1.

## 3. Results

**Figure 2:**
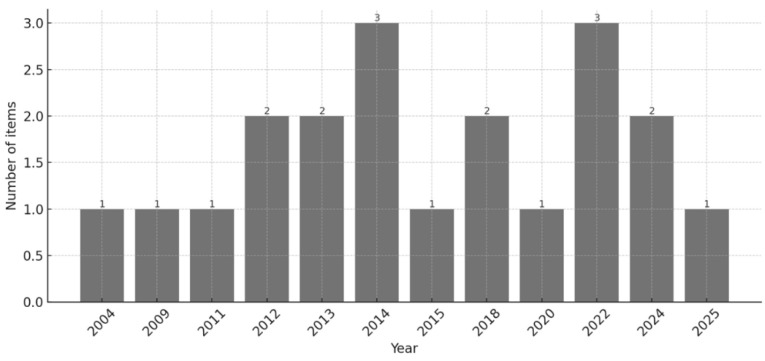
Temporal distribution of included studies.

The initial literature search yielded 538 studies. After removing duplicates, records were screened by title and abstract, followed by full-text review to assess their relevance and eligibility in relation to the research sub-questions. The full-text reading led to the inclusion of 20 items: n = 12 (60%) journal articles and n = 8 (40%) conference abstracts. Despite the absence of time restrictions in the search strategy, all included studies were published from 2004 onward. In fact, while only two studies were published before 2010 [24,42], most of them were published between 2012 and 2025, with notable peaks in 2014 and 2022 (Figure 2).

**Figure 3:**
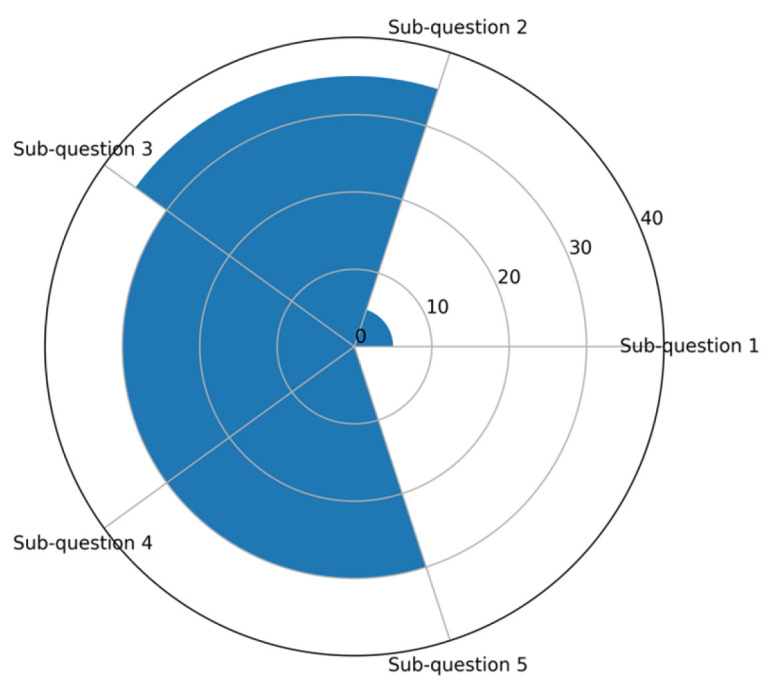
Distribution (%) of included studies across the five review sub-questions using a radar bar chart: the radar chart illustrates the proportion of included studies addressing each review sub-question. Each axis represents one sub-question, and the corresponding value indicates the percentage of studies that reported findings relevant to that thematic area. Higher values reflect greater coverage of the topic across the included literature.

Studies were conducted in hospital and academic centers across Italy and spanned multiple Italian regions. Specifically, studies were conducted primarily in Northern Italy (n = 11; 55%), followed by Southern Italy (n = 5; 25%) and Central Italy (n = 2; 10%). Two studies were multicenter (n = 2; 10%), while the remaining ones were conducted in one center (n = 18; 90%). Almost all included studies adopting a quantitative research design. No randomized controlled trials (RCTs) were identified. Most studies employed observational or descriptive designs. Two studies had a qualitative methodology (n = 2; 10%) [43,44]. Based on their primary focus, the included studies were distributed across the five sub-questions as follows: sub-question 1, one study (5%) [45]; sub-question 2, 7 studies (35%) [44,46,47,48,49,50,51]; sub-question 3, 6 studies (30%) [43,52,53,54,55,56]; sub-question 4, 6 studies (30%) [24,42,57,58,59,60] and sub-question 5, no studies (0%), as shown in Figure 3. Overall, the greatest thematic emphasis was placed on the individual dimension, focusing on Italian AYAs’ lived experiences, emotional challenges, and psychological disorders during the transition from pediatric to adult healthcare services (sub-question 2).

### 3.1. Sub-Question 1: Focus on the Healthcare Transition Process in Italy and the Profiles of AYAs Involved

With respect to the first research sub-question, evidence mainly comes from the TransiDEA study, a national Italian initiative assessing the feasibility and implementation of transition programs for AYAs with chronic conditions, including T1DM [45]. The study involved surveys of Pediatric and Adult Diabetes Centers (PDCs and ADCs), covering 93 centers. Participation was voluntary, with 75% of PDCs and 31% of ADCs agreeing to take part. Findings highlighted a fragmented scenario: 78% of PDCs and 64% of ADCs reported having a dedicated diabetes team, while 72% of PDCs and 58% of ADCs reported a formal transition protocol. Focusing on the sociodemographic and clinical characteristics of the AYA populations, beyond the TransiDEA study, which involved 41,762 participants [45], the other included studies reporting heterogeneous sample sizes, which ranged from small cohorts (n = 22 in Montali et al., 2022) [43] to several hundred (n = 223 in Maiorino et al., 2018) [54]. Where reported, both genders were represented across studies, with a slight male predominance in most cohorts. In all studies, the mean age of AYAs was ≥18 years, ranging from 18 years [51] to 28 years [59]. Age ranges consistently covered AYAs, aligning with the focus on transfer to adult services. Across studies reporting diabetes duration (DD), mean values ranged from about 9 years [53] to about 18 years [59] at the time of transition/enrollment. Participants were AYAs with long-standing T1DM. In most samples, disease duration exceeded a decade, indicating that transition typically follows an extended clinical history. Notably, no study included newly diagnosed or recent-onset cases.

### 3.2. Sub-Question 2: Focus on the Italian AYAs’ Experiences, Emotional Challenges and Psychological Disorders Related to the Transition Process

Referring to the second research question, Italian studies indicate that the transition from pediatric to adult diabetes care in AYAs with T1DM is often perceived as challenging, despite generally favorable clinical conditions. The TransiDEA study surveyed 52 young adults across five Italian diabetes centers and found that most participants (73%) had no acute complications and over half (53%) had optimal metabolic control before transition, yet around 40% reported difficulties, feelings of abandonment, and the need for better support and communication [44]. Emotional distress, anxiety, and fear of leaving pediatric care were also documented in the Verona Diabetes Transition Project [51], where girls reported higher depressive symptoms, disordered eating behaviors, and worse emotional states than boys, with mood and HbA1c worsening by transition. Zito et al. (2011–2013) found that younger adolescents exhibited more adaptive coping strategies, whereas older youth showed more maladaptive psychological profiles [46,47,48]. While most patients (80%) felt adequately prepared and satisfied with adult services, many (62%) did not view transition as a meaningful step toward adulthood and preferred pediatric care [49]. One study describing the long-term follow-up of 69 individuals transferred at a mean age of 23.8 years, now with a mean age of 34, revealed persistent psychological issues, including depression (11.2%) and substance misuse (5.6%), highlighting the need for ongoing multidisciplinary support throughout and beyond the transition period [50].

### 3.3. Sub-Question 3: Focus on How Technology and Medical Devices Influence the Transition Process

With respect to the third sub-question, the results highlight the advantages of diabetes-related technologies in the management of T1DM among AYAs during the transition period. The use of insulin pump therapy for continuous subcutaneous insulin infusion (CSII) was consistently associated with improved glycemic control and reduced glycemic variability, as well as greater treatment satisfaction [43,52,53,54,55,56]. Maurizi et al. (2018) [55] and Pasquini et al. (2022) both reported a growing use of diabetes technologies during the transition from pediatric to adult care, with increased adoption of CSII [55] and Continuous Glucose Monitoring (CGM)/Flash Glucose Monitoring (FGM) associated with improved glycemic control in young adults with T1DM [56].

### 3.4. Sub-Question 4: Focus on Strategies, Models or Interventions for Transitional Care and Related Outcomes of Transition Process in Italy

In relation to the fourth research question, few structured transition programs for Italian AYAs with T1DM have been reported, and these generally demonstrated better clinical outcomes compared to unstructured or routine pathways [24,42,57,60]. The transition models identified differ in their structure, delivery modalities, and composition of the multidisciplinary team. In some studies, the transition process focused on a multidisciplinary team including a physician, nurse, nutritionist, and psychologist [57,60], while other models, such as that described by Cadario and colleagues (2009), relied on a transition coordinator, typically a pediatric diabetologist, to ensure continuity of care with the adult endocrinologist [24]. In a few reports, the transition process involved a transfer between departments within the same hospital [42], whereas in others, it entailed a change in healthcare facility or in the local health service responsible for patient care [59]. Moreover, indicators of successful transition varied widely and included organizational criteria such as attendance at adult clinic visits, with Agosti and colleagues (2015) reporting that nearly a quarter of patients failed to attend follow-up [58]. Other measures included biochemical parameters, particularly HbA1c, and patient-reported outcomes and experience measures reflecting satisfaction with the transition process. The timing of outcome assessment also varied, with most studies evaluating outcomes near the time of transfer and only a few including long-term follow-up. Rollo and colleagues (2014) reported no overall change in HbA1c eight years after transition, suggesting that glycemic control tends to “track” from adolescence into adulthood, with higher HbA1c at transfer predicting later drop-out [50]. Pieralice and colleagues (2020) investigated post-transition outcomes stratified by adult care center, insulin regimen, and age at transition [59]. They observed a rapid improvement in metabolic control after transition, independent of referral center, insulin regimen, or age at transfer, highlighting the potential benefit of structured educational programs [59].

### 3.5. Sub-Question 5: Focus on Assessment Criteria and Availability of Italian Tools for Evaluation of Transition Readiness

The fifth sub-question, aimed at reviewing the assessment criteria and availability of Italian tools for evaluating transition readiness, highlighted the lack of validated instruments for assessing preparedness for transition in the Italian context.

Table 1 provides a summary of the main characteristics and key findings of the studies included in this scoping review.

## 4. Discussion

In Italy, epidemiological trends document a growing number of children and adolescents developing T1DM. This pattern reflects an urgent and progressively increasing need to address the healthcare requirements of affected AYAs, who must navigate the transition from pediatric care systems to adult healthcare services. A review of the existing literature on this topic identified 20 studies from the Italian context published over the past two decades, characterized by substantial heterogeneity in study design, methodologies, and research objectives.

### 4.1. Sub-Question 1: Focus on the Healthcare Transition Process in Italy and the Profiles of AYAs Involved

The first sub-question was primarily addressed by a national survey, the TransiDEA study [44], also if lower participation of the ADCs potentially could introduce selection bias and underrepresent some settings. However, the remaining studies included in this scoping review were highly heterogeneous, both in terms of sample size, ranging from small cohorts to several hundred participants, and with respect to sociodemographic characteristics. Sex distribution was generally balanced, with a slight male predominance. Nevertheless, some studies did not report sex, underscoring the need for a minimal dataset that systematically includes sex/gender information to support gender-responsive analyses. Participant age profiles reflected the transition window: mean ages were consistently ≥18 years, aligning with European practices. An International Society for Pediatric and Adolescent Diabetes (ISPAD) survey showed that 76% of centers follow young people up to age 18, although transition ages vary widely, ranging between 14 and 25 years [61]. Beyond sociodemographic characteristics, from a medical and anamnesis perspective, in the included studies, diabetes duration ranged from 9 years [53] to 18 years [59] at transition/enrollment. No studies included newly diagnosed or recent-onset cases, likely to avoid confounding related to the “honeymoon phase,” a transient period after T1DM diagnosis characterized by partial β-cell recovery, reduced insulin requirements, and temporarily improved glycemic control. This age–duration pairing suggests that transitions often occur relatively late, warranting investigation into structural, behavioral, and sociocultural factors that delay transfer. Consistent with this, a recent retrospective cohort study reported that older age at the last pediatric visit and urban residence increased the likelihood of timely transition, although the authors acknowledged limitations, including potential unmeasured confounding and limited generalizability beyond universal-coverage health systems [62].

### 4.2. Sub-Question 2: Focus on the Italian AYAs’ Experiences, Emotional Challenges and Psychological Disorders Related to the Transition Process

Regarding the second research question, studies from the Italian context indicate that the transition from pediatric to adult diabetes care in AYAs with T1DM is often perceived as challenging, despite generally favorable clinical conditions at the time of transfer. Montali et al. (2022) conceptualized patients’ experience as a lifelong journey, beginning with a difficult onset at diagnosis and continuing through identity integration and the progressive acquisition of expertise [43]. Other Italian authors have also highlighted the emotional and psychological difficulties associated with transition, emphasizing the need to address patients’ fears, uncertainties, and emotional distress related to leaving pediatric care [44,46,47,48,49,50,51]. Evidence from the TransiDEA study and other Italian investigations highlights frequent emotional distress, anxiety, and feelings of abandonment, alongside increased autonomy following transfer [44]. Psychological difficulties tend to intensify approaching the transition, with female patients appearing particularly vulnerable. Although most patients report adequate preparation and satisfaction with adult services, many do not perceive transition as a positive developmental milestone and maintain a strong attachment to pediatric care. Long-term follow-up data suggest that inadequate transitional support may be associated with persistent psychological distress in adulthood [50]. These findings align with international literature. A recent qualitative meta-synthesis showed that adolescents with T1DM navigate highly complex emotional and experiential trajectories during the transition to adult care [63]. This process is often marked by significant diabetes distress, defined as the hidden emotional burden associated with ongoing self-management demands, including frustration, hopelessness, anger, guilt, and fear.

Diabetes distress affects up to 67% of adolescents with T1DM and can negatively impact self-management behaviors and glycemic control [64,65]. In some cases, it may evolve into clinically relevant psychological or psychiatric conditions. A systematic review and meta-analysis confirmed a high prevalence of depressive and anxiety symptoms in youth with T1DM, particularly during vulnerable life stages such as the transition to adult care, potentially compromising both diabetes management and metabolic outcomes [66]. These findings support recommendations for early screening and regular psychosocial assessment from diagnosis, with follow-up continuing across the lifespan. In line with this, Rollo et al. (2014) assessed long-term well-being and clinical stability eight years after transition to adult care, and their results are consistent with international evidence [50]. The study highlights the potential long-term impact of transitional care on mental health and underscores the importance of comprehensive, multidisciplinary follow-up extending well beyond the immediate post-transition period [67]. Notably, diabetes distress and transition readiness appear inversely related, suggesting a bidirectional interaction in which emotional burden and preparedness for adult care mutually influence each other. The directionality of this relationship, however, remains unclear, highlighting the need for prospective studies to clarify causal pathways and inform evidence-based, integrated transition care models.

### 4.3. Sub-Question 3: Focus on How Technology and Medical Devices Influence the Transition Process

The importance and role of technological tools and medical devices for blood glucose monitoring and insulin administration during the transition from pediatric to adult care have been reiterated in several Italian studies [52,53,54]. Overall, the findings indicate that diabetes technologies play a beneficial role during transition in AYAs with T1DM. At the international level, a recent systematic review and meta-analysis found that, compared with other insulin regimens or standard care, the use of an automated insulin delivery (AID) system for more than six months in youth with T1DM was associated with clinically meaningful improvements in multiple measures of glucose management, particularly during nighttime, and with a reduced risk of both hyperglycemia and hypoglycemia, without an increase in adverse events [68]. However, the effects of AID systems on quality of life remain unclear, and more data are needed on their efficacy for patient-reported outcomes [68]. Despite the increasing number of technological devices on the market and advances in telemedicine modalities available to patients with T1DM, the utilization of technology remains suboptimal among patients at transition age [69,70]. This critical issue can also be contextualized in the Italian setting, as documented by the Working Group on Diabetes and Technology AMD–SID–SIEDP [71]. This study showed that, although the use of CSII and CGM increased in Italy between 2013 and 2018, the proportion of users is still lower than expected based on clinical indications for technology use: the percentages of adult and pediatric patients with T1DM on CSII were 21% and 32%, respectively, and 35% and 57% for CGM [71]. It is important to emphasize that technology use is not automatic and should be integrated within a shared, continuously updated education program. A qualitative study with 22 patients highlighted that technology can act both as a facilitator and a barrier to self-care [43].

### 4.4. Sub-Question 4: Focus on Strategies, Models or Interventions for Transitional Care and Related Outcomes of Transition Process in Italy

In relation to the fourth research question, which focused on identifying strategies, models, and interventions for transitional care implemented or tested at the national level, available evidence suggests that structured, multidisciplinary transition programs may improve clinical outcomes among Italian AYAs with T1DM [24,42,57,58,59,60]. However, heterogeneity in program design, team composition, and outcome measures limits comparability across studies. These findings are consistent with international literature demonstrating the benefits of structured transition programs in improving clinical outcomes [72]. Such programs typically provide structured support, including self-care training, case management, and access to digital tools to actively prepare adolescents and young adults for transition [73]. Although several transitional care strategies have been described, comprehensive data on transition programs for Italian youths with T1DM remain limited, and variability in both the type and timing of outcome measures complicates systematic evaluation and hinders direct comparison across structured transition programs [60].

### 4.5. Sub-Question 5: Focus on Assessment Criteria and Availability of Italian Tools for Evaluation of Transition Readiness

Assessing transition readiness is a crucial step in the transition process, and suitable readiness measures are necessary to facilitate effective planning [74]. These instruments should be used to determine the level of readiness and to guide educational interventions in a measurable and comparable way. The importance of tools for evaluating transition readiness in AYAs with chronic conditions is well established in the literature [32], and this aspect is particularly relevant in the context of T1DM, a condition whereby management relies heavily on adherence to daily self-care behaviors [75]. Unlike many chronic conditions where the consequences of poor adherence develop gradually (i.e., hypertension or asthma), in T1DM, management errors or omissions may lead to immediate and potentially life-threatening complications, such as diabetic ketoacidosis or severe hypoglycemia. Research in this field is rapidly evolving, and several diabetes-specific readiness studies have been published recently [76]. However, findings from this scoping review indicate that none of the identified tools have been validated for use in the Italian context. The literature included in this analysis shows a strong emphasis on the structural aspects of transition, such as how programs should be designed, the optimal timing for transfer, and the healthcare professionals involved, yet a clear gap remains regarding how and when adolescents’ readiness should be assessed. Only a limited number of studies have addressed this aspect. Some evidence points to the importance of shared evaluation criteria. For example, Pasquini et al. (2022) reported that transfer to adult care occurred only when complete agreement was reached among all members of the multidisciplinary team [60]. This finding underscores the need for further research aimed at developing structured and consensus-based approaches to transition readiness assessment, incorporating not only the perspectives of healthcare professionals but also those of adolescents and their families. A coordinated and multidimensional assessment process would be crucial for ensuring successful and individualized transitions.

## 5. Conclusions

This scoping review highlights the growing attention over the past decade to the transition from pediatric to adult care for young people with T1DM in the Italian context. Although several transition models and strategies have been implemented, the available evidence remains fragmented and methodologically limited, with a predominance of single-center observational studies and the absence of randomized controlled trials. Overall, structured transition programs appear to be associated with improved clinical outcomes and higher patient satisfaction. However, substantial variability persists in team composition, organizational processes, and timing of transition. A major gap identified concerns the assessment of transition readiness, which is infrequently standardized and rarely supported by validated instruments available in Italian. In addition, the psycho-emotional dimensions of transition and its long-term impact on treatment adherence and mental health are insufficiently explored. While the use of diabetes technologies shows promise in fostering autonomy and improving metabolic control during transition, their uptake and integration into care pathways remain inconsistent. Based on these findings, several actionable recommendations emerge. Future research should prioritize high-quality, multicenter studies capable of capturing the heterogeneity of the national context and ensuring adequate sample sizes, with stratified analyses by age group (adolescents versus young adults) and gender to better reflect developmental and psychosocial differences. There is a clear need to develop and implement culturally and linguistically validated Italian tools for assessing transition readiness, which would support more individualized and effective care planning. Furthermore, the implementation of structured, multidisciplinary transition programs, alongside age- and gender-sensitive approaches to transitional care, represents a critical step toward optimizing outcomes for young people with T1DM moving into adult healthcare services.

## Figures and Tables

**Table 1:** Characteristics and key findings of included studies.

Authors, Year	Geographical Area in Italy	Type of Article	Study Design	Aim	Population (N: Gender ^1^; Years Old: Mean ± SD; Years Old at DO: Mean ± SD; DD: Mean ± SD)	Main Findings/Results
Vanelli et al., 2004 [42]	North (Parma)	Academic paper	Retrospective observational study	To describe the effects of an 8-year-long uninterrupted transition procedure of adolescents from pediatric to adult clinic in the same hospital	73 subjects: 21.0 ± 0.95 yrs at transition	Patients expressed high satisfaction with the transition process. All participants (100%) felt well-informed beforehand, and 92% appreciated meeting the adult care physician prior to transfer. The presence of their pediatrician during the first adult clinic visit was valued by all (100%). For most patients (66.6%), a shared decision was reached after two to four consultations. The majority (79%) considered age 20 appropriate for transfer. Upon entering adult care, patients highlighted several positives (78–100%): adequate privacy; trusted confidentiality; appreciated short waiting times; welcoming, informal environment; to be followed by the same consultant. Only 3% considered returning to pediatric care but were dissuaded. Overall clinic attendance: 92% to 100%.
Cadario et al., 2009 [24]	North (Novara)	Academic paper	Descriptive retrospective study	To evaluate and compare a structured transition from the PDS into the ADS with an unstructured one	62 subjects: 19.0 ± 2.8 yrs	Patients who underwent a structured transition (Group B) had a significative shorter transfer time, better clinical attendance, and significantly lower HbA1c values both at the first visit and after one year in adult care compared to those who experienced an unstructured transition (Group A). Moreover, all subjects in Group B reported a favorable opinion of the structured process (*p* < 0.0001). Lack of medical assistance during the transition was a critical issue in Group A (*p* < 0.001).
Zito et al., 2011 [46]	South (Naples)	Conference abstract	Descriptive study	To psychologically evaluate adolescents and young adults still followed at the Department of Pediatrics who had to move to adult care units	105 subjects Group 1: 77 Adolescent; M = 48.5% 18.6 ± 1.6 yrs. Group 2: 28 Young Adults; M = 35.7%; 23.3 ± 1.7 yrs	Both groups expressed strong attachment and trust in their pediatricians (85–87%) 87% in Group 1, 85% in Group 2), along with fears related to separation from the pediatric setting and encountering new healthcare providers. Group 1 showed more adaptive psychological responses, including healthier defense mechanisms and better therapeutic compliance, compared to Group 2. No significant psychopathological symptoms were identified in either group (SCL-90 screening).
Zito et al., 2012 [47]	South (Naples)	Conference abstract	Descriptive study	To psychologically evaluate patients in transition	113 subjects Group A: 83 Adolescents; M = 49.4%; 18.6 ± 1.6 yr; DO: 8.9 ± 4.6 yr Group B: 30 Young Adults: M = 36.7%; 23.8± 2.2 yr; DO: 8.9 ± 4.2 yrs	The REM-71 results indicated that Group A exhibited more adaptive and functional psychological defense mechanisms in response to chronic illness compared to Group B (*p* < 0.005). Similarly, the CIDS scores revealed higher levels of treatment adherence in Group A than in Group B (*p* < 0.005). No significant psychopathological symptoms were identified in either group based on the SCL-90-R. In both groups, a strong fear of separation from pediatric providers and anxiety about transitioning to adult care were commonly reported.
Aglialoro et al., 2012 [49]	North (Genoa)	Academic paper	Descriptive study	To assess youths’ satisfaction with a structured transition using the “Transition Satisfaction questionnaire” recommended by the SIEDP–AMD–SID Consensus Study Group	56 subjects: M = 58.9%; 27.5 ± 11 yrs at transition time	Most of the patients referred anxiety disorders related to transition from PDC to ADC (93%), recognized an appropriated planned training to transition (80%), and were satisfied about the new diabetologic adult team efficiency (welcome, take care of patients, treatments, global information, waiting period) (6 questions: 78–90%). Nevertheless, 62% did not see transition as beneficial, and 64% would return to pediatric care.
Grassi et al., 2013 [52]	North (Turin)	Academic paper	Observational longitudinal cohort (retrospective follow-up)	To describe and evaluate the metabolic and adherence outcomes on CSII of a “out-patient transition–technology” program from PDS into the ADS	98 subjects: 18.5 ± 5.7 yrs at first observation. Divided into two homogeneous groups	HbA1c remained stable over a mean follow-up of 5.6 years. Two years post-transition, metabolic control was preserved (HbA1c: 8.60% → 8.23% → 8.35%). Drop-out from CSII was 10.2%, while only one patient (2%) left the ADC. These findings suggest that late adolescence is a favorable period for structured transition, and that CSII supports therapeutic continuity without compromising glycemic outcomes.
Zito et al., 2013 [48]	South (Naples)	Conference abstract	Descriptive study	To psychologically evaluate patients in transition in order to create an efficacious care pathway to accompany them	118 subjects Group A: 85; M = 49.4%; 18.7 ± 1.5 yrs; DO: 8.9 ± 4.5 yrs. Group B: 33; M = 42.4%; 23.8 ± 2.2 yrs; DO: 8.9 ± 4.1 yrs	Group A exhibited more adaptive psychological defenses (REM-71) and better diabetes care compliance (CIDS) than Group B. No psychopathological traits were detected in either group (SCL-90-R). Older patients showed more dysfunctional profiles, while most participants expressed strong separation anxiety from pediatricians and apprehension toward adult care providers.
Rollo et al., 2014 [50]	North (Bologna)	Academic paper	Prospective observational study	To assess the relationship between pediatric glycemic control and adult outcomes, and to evaluate the prevalence of complications, comorbidities, and psychological or psychiatric disorders in a patient cohort, approximately 8 years post-transition to ADC	69 subjects: M = 46%; Current age 34.1 ± 4.6 years; DO: 8.4 ± 3.8 years; Age at transition 23.8 ± 3.9 yrs	Mean HbA1c remained stable across pediatric, transition, and adult phases [8.4 ± 1.8%, 8.3 ± 1.4%, and 8.4 ± 1.3%, respectively]. Thirteen patients dropped out 2–12 years post-transition, with a mean HbA1c of 10.4% at transition. After a mean of 25.9 years of disease, 50.7% developed retinopathy and 17.3% nephropathy. The most frequent comorbidities were thyroid disorders (18.3%), depression (11.2%), and benign neoplasms (9.8%). Substance abuse was reported in 5.6% of cases. Poor metabolic control at transition was associated with increased risk of drop-out and psychosocial morbidity.
Da Porto et al., 2014 [57]	North (Trieste)	Conference abstract	Descriptive study	To examine the impact of structured multidisciplinary education during transition on post-transition glycemic control.	55 subjects: 27.8 ±10.1 yrs; DD: 17.3 ± 9.9 yrs	During a 6.2 ± 9.1 month transition gap, HbA1c worsened by +0.32%. At the first adult visit, HbA1c averaged 7.9%, with high glycemic variability (83.1 mg/dL) and 12.6% hypoglycemia. Initially, only 23.6% applied CHO correctly and 11% followed a constant CHO diet. After one year, these improved to 34.6% and 25.2% (*p* = 0.014, 0.037). HbA1c dropped by −0.72% (*p* = 0.009), variability by −12% (*p* = 0.041), while hypoglycemia reduction was not significant. CHO management showed benefits in univariate analysis but not in multivariate. Baseline HbA1c was the only significant predictor of 12-month glycemic control (R = 0.0659, *p* = 0.0001).
Maiorino et al., 2014 [53]	South (Naples)	Academic paper	Observational study	To evaluate whether CSII may have any advantage over MDI on glycemic control and treatment satisfaction in young patients in transition from PDC to an ADC	120 subjects Group CSII: 38; M = 60% 21.7 ± 2.3 yrs; DD: 9.1 ± 4.0 yrs Group MDI: 82; M = 59.7%; 21.4 ± 1.9 yr; DD: 10.2 ± 4.0 yr.	Among patients transitioning from PDC to ADC, CSII demonstrated comparable efficacy to MDI in reducing HbA1c while offering greater benefits in lowering glycemic variability and overall hypoglycemia. CSII was also associated with higher treatment satisfaction and improved perception of both hyperglycemia and hypoglycemia.
Agosti et al., 2015 [58]	North (Brescia)	Academic paper	Retrospective observational study	To describe the transition of adolescents from PDC to ADC, highlighting critical issues and reasons for drop-outs	83 subjects: M = 45.7%; 19.1 ± 1.5 yrs	Twenty-two percent missed post-transition follow-up. In multivariable analysis, continuity with the same physician during the first year was the only significant protector against drop-out (OR 0.352; 95% CI 0.161–0.645; *p* < 0.0001).
Maiorino et al., 2018 [54]	South (Naples)	Academic paper	Observational study	To evaluate the long-term effects of CSII therapy, compared with MDI, on GV in patients with suboptimal glycemic control, transitioned to the ADC	223 patients completed the 2-year follow-up: Group CSII: 98; M = 52.1%; 25.3 ± 3.3 yrs; DD: 14.2 ± 4.9 yrs. Group MDI: 125; M = 61.6%; 24.5 ± 2.9 yrs; DD: 13.7 ± 4.1	The use of CSII was associated with a greater reduction in GV, fasting glucose levels, and total insulin dose compared to MDI, despite achieving similar improvements in HbA1c over a two-year follow-up. Additionally, CSII proved more effective in reducing the incidence of hypoglycemia, daily, nocturnal, and severe, and in improving the perception of hypoglycemic episodes.
Maurizi et al., 2018 [55]	Center (Rome)	Conference abstract	Descriptive study	To assess the metabolic status of patients during the transition from PDC to ADC, following international consensus guidelines (“transition clinic” based on the protocol of the Consensus Statement of the American Academy of Paediatrics, American Diabetes Association, Academy of Family Physicians and the American College of Physicians)	122 subjects: M = 67.2%; 25.1 ± 5.7 yrs; DD 17.2 ± 8.1 yrs	Significant reductions in HbA1c were observed at 3 and 6 months post-transfer (−0.3%, *p* < 0.05; −0.5%, *p* < 0.02) across all age groups. Female patients had worse glycemic control than males at both baseline and follow-up (*p* = 0.005 and *p* < 0.001, respectively). CSII use increased from 16% to 27% post-transition, but HbA1c improvement was independent of insulin delivery method.
Pieralice et al., 2020 [59]	Center (Rome)	Conference abstract	Retrospective observational study	To evaluate whether metabolic control after transition differs by different adult care center, insulin regimens and age at transition	178 subjects: 28.4 ± 6.7 yrs; DD: 18.6 ± 8 yrs	This study demonstrates a prompt improvement in metabolic control among individuals with T1D following the transition to ADC, independent of the referral center, insulin therapy type, or age at transition. Further research is warranted to assess the impact of various educational programs on post-transition outcomes.
Pasquini et al., 2022 [56]	North (Verona)	Conference abstract	Prospective observational study	To assess technology use in emerging adults belonging to the VDTP	161 subjects: M = 53.4%; 24.8 ± 6.1 yrs; DD: 14.5 ± 6.9 yrs	Technology use rose from 19% to 65%, mainly CGM/FGM (from 14% to 62%), which was linked to lower poor diabetes acceptance (*p* = 0.02). After 36 months, HbA1c reduced (from 8.31% to 7.42%, *p* < 0.001), TIR increased (50.1% to 59.4%, *p* < 0.001), TAR > 250 and TBR < 54 decreased (*p* = 0.008, 0.003), and CV dropped (from 41.8% to 38.3%, *p* = 0.005). Regression showed sensor use predicted lower HbA1c (βst = −0.24, *p* = 0.02), while fear of hypoglycemia predicted higher HbA1c (βst = 0.23, *p* = 0.03).
Montali et al., 2022 [43]	North (Monza)	Academic paper	Qualitative study	To explore the lived experience of adolescents and young adults, with a particular focus on self-care practices, barriers and facilitators	22 subjects: M = 32%; 21.5 yrs; DD: 13 yrs	Living with T1D is a lifelong process that begins at diagnosis and progresses through identity development and the acquisition of self-management skills. Both technology and the social environment serve as facilitators and obstacles to optimal care. To enhance clinical outcomes, diabetes technologies must be intuitive, reduce stigma, and minimize treatment burden. Healthcare providers should address the psychosocial aspects of T1D, particularly during the pediatric-to-adult care transition, through comprehensive, person-centered assessments.
Pasquini et al., 2022 [60]	North (Verona)	Academic paper	Prospective observational study	To identify clinical, socio-demographic, and psychosocial factors associated with glycemic control of youth at the time of transition to ADC attendance	222 subjects: M = 50.9%; 24.4 ± 5.8 yrs at transition; DD: 14.4 ± 6.6 yrs	Women showed higher HbA1c values (70 ± 11 mmol/mol vs. 65 ± 7 mmol/mol or 8.57% ± 1.51% vs. 8.14% ± 0.98%, *p* = 0.01), higher frequency of disorders of eating behaviors (15.6% vs. 0%, *p* < 0.001) and poor diabetes acceptance (23.9% vs. 9.7%, *p* < 0.001) than men. Mediation analyses showed a significant mediating role of glucose control 2 years before transition in the relationship between poor diabetes acceptance and glucose control at transition.
Fasoli et al., 2024 [51]	North (Verona)	Conference abstract	Prospective observational study	To explore the psychosocial factors in youths consecutively enrolled in the VDTP	77 subjects: M = 49.3%; 18 yrs; DD: 9.9 ± 3.8 yrs	At baseline, females exhibited significantly higher scores in depressive symptoms, eating disorders, body dissatisfaction, and emotional distress compared to males (all *p* < 0.001). After two years, at age 18, mood disturbances worsened (mean score: 44.6 ± 10.3 vs. 50.7 ± 10.0; *p* = 0.01) and glycemic control declined (HbA1c: 7.3 ± 0.8 vs. 7.7 ± 1.1; *p* = 0.02). These findings underscore the progressive emotional burden experienced by adolescents in the VDTP and highlight the need for sustained psychological support throughout the transition process.
Graziani et al., 2024 [45]	Italy	Academic paper	National survey	To evaluate how the transition process was managed throughout ADCs and PDCs and to understand the current state of Italian assistance.	41.762 subjects: minors (age < 18 years) = 85%; young adults (age > 18 years) = 15%	The survey revealed variability in transition practices. A dedicated diabetes team was present in 78% of PDCs and 64% of ADCs. Transition protocols were reported by 72% of PDCs and 58% of ADCs. The median transition age was 19 years (range: 16 to 25), with a preparation period of approximately 5.5 months. While 80% of ADCs reported receiving adequate clinical information, primarily via paper or digital formats, the transition process remains hindered by limited resources, inadequate infrastructure, and poor inter-service communication.
Graziani et al., 2025 [44]	Italy	Academic paper	Qualitative survey	To conduct a qualitative study to gather the experiences of individual patients, their families, and clinicians regarding the transition process	52 subjects: M = 42.3%; median age 23; North = 55.79% Islands = 25% South = 19.2%; 5 parents also participated.	A significant number of patients reported challenges in the operational aspects of the transition, with approximately 40% describing the experience as difficult. Some expressed feelings of being “abandoned” and emphasized the need for stronger support and better communication between pediatric and adult services. Despite these difficulties, most respondents noted a greater sense of autonomy in managing their diabetes after the transition.

**^1^ Acronyms:** ADC: Adult Diabetes Center; ADS: Adult Diabetes Service; AMD: Associazione Medici Diabetologi; CGM: Continuous Glucose Monitoring; CHO: carbohydrate counting; CI: Confidence Interval; CIDS: Confidence in Diabetes Self-Care; CSII: Continuous Subcutaneous Insulin Infusion; CV: glycemic variability; DD: Diabetes duration; DO: Diabetes onset; FGM: Flash Glucose Monitoring; HbA1c: Hemoglobin A1c; MDI: Multiple Daily Injections; M/F: Male/female; OR: Odds Ratio; PDC: Pediatric Diabetes Center; PDS: Pediatric Diabetes Service; REM-71: Response Evaluation Measure-71; SCL-90: Symptom Checklist-90; SIEDP: Società Italiana di Endocrinologia e Diabetologia Pediatrica; SID: Società Italiana Diabetologia; TAR: Time Above Range; TBR: Time Below Range; TIR: Time in Range; VDTP: Verona Diabetes Transition Project; yrs: years

## Data Availability

The data supporting the findings of this study are available from the corresponding author upon reasonable request.

## Supplementary Materials

The following supporting information can be downloaded at: https://www.mdpi.com/article/10.3390/children13020248/s1. Table S1. Example of the search strategy for MEDLINE (PubMed). Table S2. Preferred Reporting Items for Systematic reviews and Meta-Analyses extension for Scoping Reviews (PRISMA-ScR) Checklist [35].

## Abbreviations

The following abbreviations are used in this manuscript:

ADA	American Diabetes Association
ADCs	Adult Diabetes Centers
AID	Automated Insulin Delivery
AYAs	Adolescents and Young Adults
CGM	Continuous Glucose Monitoring
CSII	Continuous Subcutaneous Insulin Infusion
DD	Diabetes Duration
EURODIAB	European Diabetes Study
FGM	Flash Glucose Monitoring
GBD	Global Burden of Diseases, Injuries, and Risk Factors Study
HbA1c	Glycated Hemoglobin
ISPAD	International Society for Pediatric and Adolescent Diabetes
PDCs	Pediatric Diabetes Centers
PRISMA	Preferred Reporting Items for Systematic Reviews and Meta-Analyses
RCT	Randomized Controlled Trial
RIDI	Registro Italiano per il Diabete di Tipo 1
SID	Italian Society of Diabetology
SIEDP	Italian Society for Pediatric Endocrinology and Diabetology
T1DM	Type 1 Diabetes Mellitus
T2DM	Type 2 Diabetes Mellitus
WHO	World Health Organization
